# Reply to: The three-dimensional structure of wood enables horizontal water transport needed to conduct water around lesions

**DOI:** 10.1038/s41598-023-41785-z

**Published:** 2023-09-12

**Authors:** Lars Dietrich, Ansgar Kahmen, Günter Hoch, Christian Körner

**Affiliations:** 1 Department of Environmental Sciences, University of Basel, Schönbeinstrasse 6, 4056 Basel, Switzerland; 2 Agroecology and Organic Farming Group, Institute of Crop Science and Resource Conservation, University of Bonn, Auf dem Hügel 6, 53121 Bonn, Germany

**Keywords:** Plant ecology, Plant physiology

**replying to**: P. Hietz; *Scientific Reports* 10.1038/s41598-023-41817-8 (2023).

We are glad to see that our experiment stimulated the debate on the hydraulic significance of tree stems in the soil–plant–atmosphere continuum (SPAC). Hietz et al. provide robust theoretical guidance toward a cell-level interpretation of the results we obtained by cutting 50% of the sapwood area of tall *Picea abies* L. and *Fagus sylvatica* L. trees, with no effect on the crowns’ water status as assessed by means of a canopy crane^1^. The mathematics- and physics-based model of Hietz et al. is impressive and clearly points at a fruitful avenue for a better understanding of water flows around barriers in tree stems. Not theoreticians ourselves, we think the interdisciplinary approach by Hietz et al. holds big promises. From the results of our transverse cut experiment we concluded that at maximum summer sap flux rates, the conduits of the investigated trees operate far below their actual capacity as evidenced by a 40% (Picea) and 200% (Fagus) increase of flux rates across the intact conduits near the cut.

Although it is limited to tracheid wood, we sympathize with the approach by Hietz et al. and explicitly do not question that the rate of sap flow is controlled by water potential gradients and hydraulic resistances. We also acknowledge that by the laws of physics a 20 mm thick transverse cut through 50% of the sapwood area does not necessarily reduce stem total conductance by 50% as our results and the results from the modelling approach by Hietz et al. impressively show (see also Mackay and Weatherly^2^, who demonstrated that even multiple deep transverse cuts into the stem did not exert any effect on water potentials). To reduce the conductance of the entire stem by half, it would be necessary to remove half of the sapwood alongside the whole trunk or apply multiple cuts, since the resistance for water flow around such a single cut is not proportional to the cut stem area. While such a treatment would be difficult to perform in situ, we still think that our single cut holds implications for future tree hydraulics research since the massive horizontal flow detour we imposed, had no effect on crown water relations.

Hence, in contrast to Hietz et al., we think the stem xylem exerts a rather small fraction of the total resistance in the SPAC. As the cohesion-tension-theory predicts, water potentials within the plant are the result of a flux across resistors with atmospheric water demand (vpd) and soil water potential defining the gradient that drives the flux, regulated by stomatal conductance. Given the rather small vertical water potential gradients in trees reported in literature, the major SPAC resistors are found in the soil-to-root transition and in leaf hydraulic pathways (petiole, veins), with the stem’s xylem oversized for both emergency (wounding, pathogens, our cuts) and tree mechanics (stability).

Hietz et al. make a valid point, that a transverse cut is forcing flow tangentially (horizontally). Following this tangential pathway, the water has to pass through many more pits and resistances are estimated to be 20 times higher than axial resistances in conifer wood. As our results show, these additional resistances in the stem are negligible for adequate canopy water supply. However, we still contend with the model of Hietz et al. arriving at more negative water potentials near the cut, compared to a few meters above the cut. If correct, water flow would have to be reversed (water flowing down-rather than upwards).

In a PLC (per cent loss of conductance) context, it would be interesting to know how Hietz et al.’s model would reproduce a 50% PLC cavitation event. Their theoretical scenario implies that at least 50% of all conduits have to be cavitated alongside the whole length of the stem to reduce the conductance of the stem’s xylem by 50%. Alternatively, way more than 50% of all conduits need to be severed/cavitated in shorter segments of the stem to produce the same result. In angiosperm trees with a great variation of conduit size and diameter this might yield a completely different picture.

We think that such a disastrous spread of conduit cavitation in the stem would most likely only happen after the SPAC disintegrated upstream, i.e. at the soil-to-root interface, and thus assume that stem conduit cavitation is rather a by-product of below-ground water transport failure in trees than an ultimate cause, as was suggested by Körner^3^. However, there is evidence that terminal branchlets can cavitate fatally, while the main stem doesn’t^4^ underlining the importance of where, spatially, the capillary continuum becomes interrupted.

We think our results underline the comparatively low magnitude of the stem’s xylem resistance as compared to other hydraulic resistances in the SPAC. In accordance with the early works on SPAC (e.g.^5,6^) but also most recent discoveries (e.g.^7^) we assume that the soil-to-root transfer exerts the major resistor, with much of the stem’s diameter representing mechanical support for the ever battle for light among trees and the mechanical strain by strong wind and snow. The secondary role of conduits can explain why trees silence growth rings after a few years, with a very steep flux gradient from youngest to oldest conducting rings, and why some ring-porous taxa are using the last ring only. If there was a shortage in conducting capacity, why should the silencing of conductive rings be of any evolutionary advantage? Hence, we concur with Becker et al.^8^ that the hydraulic resistance of the rhizosphere diminishes the significance of the axial resistance. The soil-to-root part of water transport was considered central in all early attempts at quantifying constraints to plant water supply under drought^9,10^ leaving not much space for critical constraints by the axial part of the transport path as it became so popular recently. In our view, Hietz et al.’s intriguing explanation of our results awaits empirical validation in the light of these earlier works, but it does not falsify the notion of a marginal role of axial transport constraints in trees in a SPAC context.

Therefore, although this was neither a central issue in Hietz et al.’s modelling nor our stem cut experiment, we argue that a loss of the capillary continuum between soil and roots under severe drought would result in immediate stomatal closure causing fluxes to approach zero, thus marginalizing the required transport capacity of the stem xylem^3^. In such a scenario the flow resistance of the xylem should hardly play any role. If there is no flux, the resistance of the pathway does not exert any influence on water potentials. Thus, in our opinion, the primary cause for water transport failure in trees is likely to be found upstream, i.e. in the soil-to-root capillary continuum, and not within the stem’s hydraulic properties.

In conclusion, we believe that there is a long way to resolve the xylem’s role in a SPAC context. Although we certainly acknowledge the correlations between tree dieback events and PLC data, our impression is that the past ten years of the hydraulic failure debate rested upon the methodological ‘simplicity’ to explore stems, *versus* the difficulties in obtaining data on roots and their interaction with the soil matrix, where we expect the major innovations (see^7^).
